# The *PLOS Biology* XV Collection: 15 Years of Exceptional Science Highlighted across 12 Months

**DOI:** 10.1371/journal.pbio.3000180

**Published:** 2019-02-27

**Authors:** Lauren A. Richardson, Sandra L. Schmid, Avinash Bhandoola, Christelle Harly, Anders Hedenström, Michael T. Laub, Georgina M. Mace, Piali Sengupta, Ann M. Stock, Andrew F. Read, Harmit S. Malik, Mark Estelle, Sally Lowell, Jonathan Kimmelman

**Affiliations:** 1 Public Library of Science, San Francisco, California, United States of America; 2 Department of Cell Biology, University of Texas Southwestern Medical Center, Dallas, Texas, United States of America; 3 CRCINA, INSERM, CNRS, Université d'Angers, Université de Nantes, Nantes, France; 4 Department of Biology, Lund University, Lund, Sweden; 5 Department of Biology Howard Hughes Medical Institute Graduate Program in Microbiology Graduate Program in Biology, Massachusetts Institute of Technology, Cambridge, Massachusetts, United States of America; 6 Department of Genetics, Evolution and Environment, Center for Biodiversity and Environment Research, University College London, London, United Kingdom; 7 Department of Biology and National Center for Behavioral Genomics, Brandeis University, Waltham, Massachusetts, United States of America; 8 Department of Biochemistry and Molecular Biology, Robert Wood Johnson Medical School, Rutgers University, Piscataway, New Jersey, United States of America; 9 Center for Infectious Disease Dynamics, Departments of Biology and Entomology, The Pennsylvania State University, University Park, Pennsylvania, United States of America; 10 Division of Basic Sciences, Fred Hutchinson Cancer Research Center, Seattle, Washington, United States of America; Howard Hughes Medical Institute, Fred Hutchinson Cancer Research Center, Seattle, Washington, United States of America; 11 Section of Cell and Developmental Biology and Howard Hughes Medical Institute, University of California San Diego, La Jolla, California, United States of America; 12 MRC Centre for Regenerative Medicine, Institute for Stem Cell Research, School of Biological Sciences, University of Edinburgh, Edinburgh, United Kingdom; 13 Studies of Translation, Ethics, and Medicine, Biomedical Ethics Unit, McGill University, Montreal, Quebec, Canada

In October of 2018 *PLOS Biology* celebrated its 15-year anniversary. Our corpus includes foundational works in all aspects of the biological sciences, from cognitive neuroscience to conservation ecology. The editors, both staff and Academic, are extremely proud of the quality and breadth of science published in our journal. *PLOS Biology* has also played a pivotal role within the Open Access movement, which in the 15 years since our launch has exploded and continues to revolutionize science communication.

We commemorated our anniversary with a year-long celebration. Each month, one of our hard-working and highly-respected Editorial Board Members contributed a blog post describing their favorite *PLOS Biology* article and its impact on the respective field. Here, we collect these posts and featured manuscripts, which can also be found in this Collection [1].

These posts highlight the incredible diversity of science published in our journal. In addition to featuring our Research Articles, some of our Academic Editors chose to highlight non-standard research content. Among the mix, Piali Sengupta wrote about an important article featuring negative results, which reshaped how we think about pheromone signaling. Andrew Read discussed one of the articles published in our Magazine section, which featured emerging and forward-thinking theory on possible unforeseen outcomes of novel therapies. Jonathan Kimmelman highlighted work from our Meta-Research section on the lack of rigor by ethical oversight committees. We hope you enjoy all of the posts and we look forward to many more years of publishing cutting-edge science across all fields of biology.

## January: Cell biology by Sandra Schmid

### Watching a vesicle form

Living cells face a dilemma; in order to prevent the unregulated influx and efflux of molecules they need a plasma membrane that is literally water-tight, but they also need to be able to take up specific molecules such as proteins from their environment. One of the ways in which they solve this problem is by the use of clathrin-mediated endocytosis.

This complex and highly regulated process involves a tightly orchestrated sequence of steps that entails the formation of pits in the plasma membrane, coated with a basket-like array of clathrin (known as clathrin-coated pits, or CCPs), followed by invagination, constriction, and pinching-off to form clathrin-coated vesicles (CCVs).

This process requires not only the major coat proteins, clathrin and adaptor protein complexes (AP2) and the GTPase dynamin, but a myriad endocytic accessory proteins (EAPs), whose exact functions are still not clearly defined. Although studied for almost 50 years, the true complexity of clathrin-mediated endocytosis was only recently revealed through the advent of live cell microscopy to image the dynamics of CCPs.

For the *PLOS Biology* XV Collection I’ve chosen to highlight this article by Christien Merrifield and co-workers [2] as it described a sophisticated and highly precise microscopy-based method to detect the scission event that leads to CCV formation and maps the temporal hierarchy of EAP recruitment to CCPs.

The method involved the use of transferrin receptors externally tagged with a pH-sensitive GFP (TfR-phl); these were imaged by total internal reflection fluorescence microscopy in a perfusion chamber in which the media is periodically cycled between pH 5 and pH 7. Imaging was coordinated at 2-second intervals with each pH change, so that the appearance of pH 5-insensitive TfR-phl precisely marked the point of scission.

This elegant approach was used to comprehensively analyze the temporal hierarchy of recruitment of 34 EAPs relative to the scission event, which allowed their classification into functional modules temporally linked to CCP initiation and maturation, actin dynamics, scission, uncoating, and post-scission vesicle motility.

**Fig 1:**
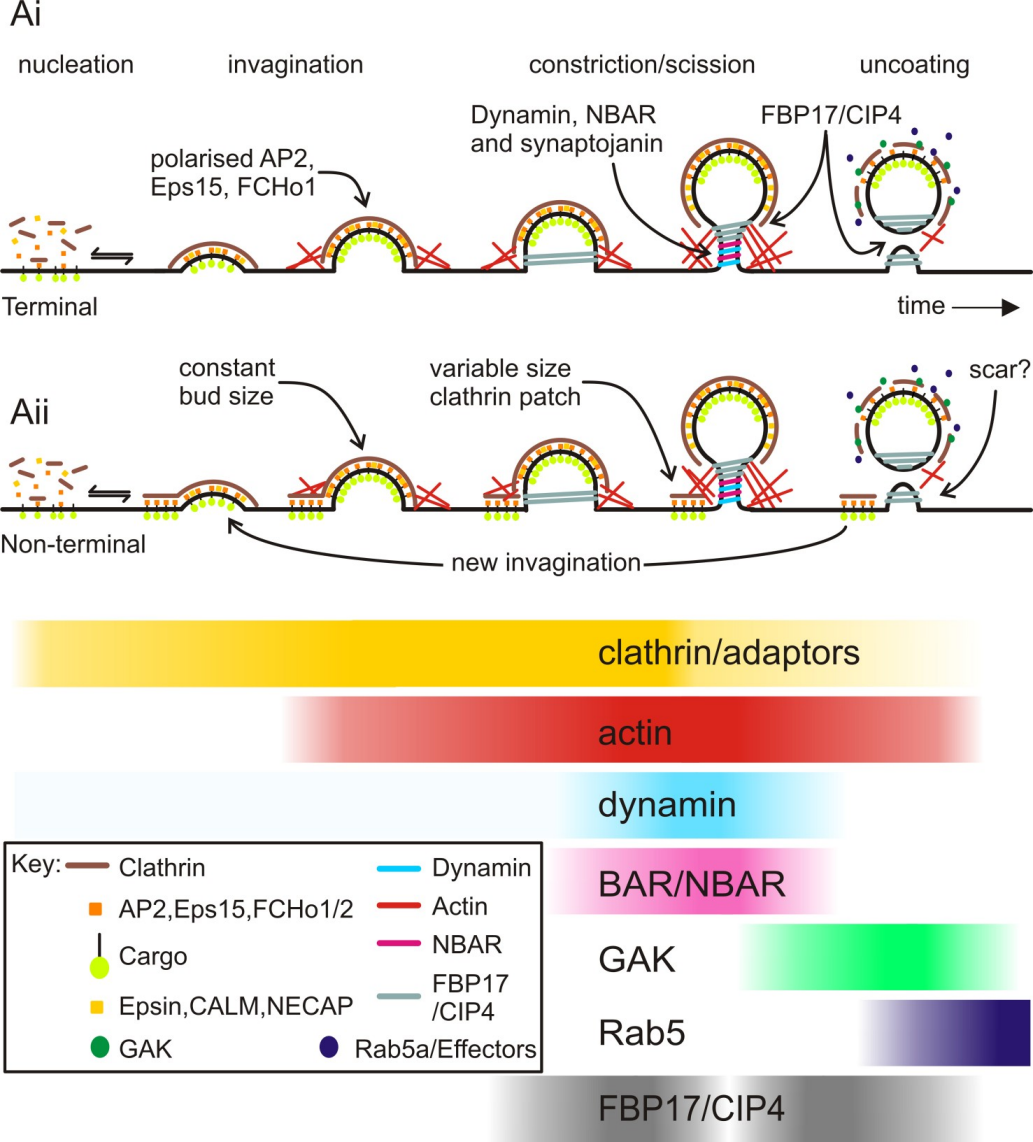
**Our view of clathrin-mediated endocytosis before (Ai) and after (Aii) Taylor et al.’s study.** Image reproduced under CC-BY 4.0 license, credit: 10.1371/journal.pbio.pbio.1000604.

The article was and remains the highest resolution temporal map of the molecular events governing the clathrin-mediated endocytosis process to date. In addition to the hierarchical classification of EAPs, the article also revealed other key principles of CCP dynamics (Fig 1).

First, the kinetics, extent and frequency of EAP recruitment to CCPs, as well as the dynamics of CCPs themselves were highly heterogenous. Merrifield reported that short-lived CCPs often failed to sequester TfR-phl and thus unambiguously established that short-lived CCPs were abortive events.

Second, in several cases Merrifield detected multiple, sequential fission events associated with continuously detected clathrin structures. These were classified as ‘non-terminal events’ that likely reflect pinching-off of CCVs from the periphery of larger clathrin-coated structures. Importantly, the recruitment signatures of EAPs to terminal vs non-terminal events were indistinguishable, suggesting a common mechanism governing CCP maturation and scission.

Third, the work established a remarkable coordination between scission and uncoating of the released vesicle.

This seminal article represents a significant leap in our understanding of vesicle trafficking and questions raised by the study continue to be addressed by researchers today. What is the significance and molecular basis for the dynamic and compositional heterogeneity of CCPs? How is uncoating so tightly coupled with membrane scission? What prevents the uncoating of deeply invaginated CCPs? What determines the productive maturation versus early abortion of CCPs?

## February: Immunology by Avinash Bhandoola and Christelle Harly

### The exquisite precision of T cell receptors

The vertebrate adaptive immune system can distinguish invaders from self with exquisite precision. The T cells, their immune receptors, and the antigenic ligands involved in this process are well characterized, but how a T cell receptor (TCR) can distinguish between closely related ligands, detect minute amounts of foreign antigens, and in turn trigger distinct downstream signals, remains poorly understood. Through its TCR, a T cell can distinguish between self and non-self ligands that differ by just a single amino acid, and T cells can be activated by a single non-self peptide that might appear to be otherwise lost among millions of self molecules. Understanding this remarkable discrimination is a major challenge in immunology.

**Fig 2:**
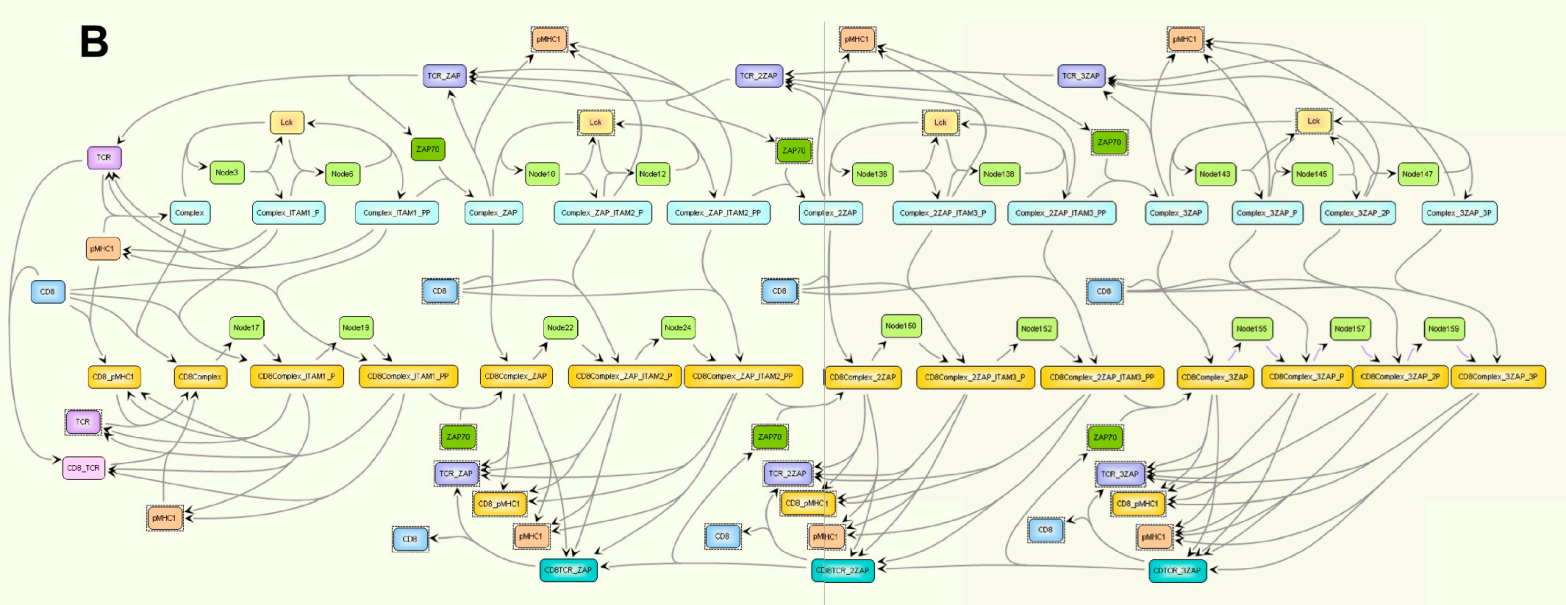
The model used by Altan-Bonnet and Germain of the core module of early events of TCR signaling. Image reproduced under CC-BY 4.0 license, credit: 10.1371/journal.pbio.0030356.

In the foundational article that we have chosen to highlight for the *PLOS Biology* XV Collection, Grégoire Altan-Bonnet and Ron Germain addressed this problem [3]. Using quantitative measurements and mathematical models, they showed that the previously well-established kinetic-proofreading model could not adequately explain the exquisite discrimination that T cells are capable of. To improve upon it, they incorporated into this model a negative feedback pathway previously suggested to sharpen the discrimination threshold between closely related TCR ligands. The new model accurately predicted the behavior of T cell activation in response to different TCR ligands, and accounted for the speed, sensitivity, and specificity of TCR-dependent activation (Fig 2).

This model depicts the TCR signaling pathway as a tunable switch. The switch is provided by two discrete states of ERK phosphorylation that the authors document for the first time and propose to be an early correlate for T cell activation. The sensitivity and specificity of this switch is tuned via the negative feedback loop by molecular players whose activity could be set during development, and further modulated by additional signaling pathways downstream of other receptors on T cells.

The work also provides an explanation for the seemingly counterintuitive behavior of antagonistic TCR ligands. An agonist ligand activates a T cell response, but an antagonist ligand is one that blocks activation by an agonist ligand. Such ligands are proposed to trigger the negative feedback loop of a given TCR without reaching the threshold of activation, thus antagonizing activation by the agonist ligand. The closer a non-agonist ligand is to reaching the activation threshold, the more antagonistic it will appear. The function of antagonist ligands is still unclear, but more recent work by Paul François & coworkers [4] derived a theorem demonstrating how antagonism is in fact a phenotypic “spandrel” (Stephen Jay Gould’s term) of sharp ligand discrimination. In other terms, the evolution of the negative feedback that receptors need to achieve the necessary sensitivity and specificity also led to the appearance of antagonism.

The model developed by Altan-Bonnet and Germain, which is an “adaptive kinetic proofreading” model, has endured the test of time. The negative feedback could apply to any receptor and in fact could be a general scheme for any complex system performing a classification task such as self/non-self discrimination in the adaptive or innate immune systems. Hence, this study focused on TCR signaling has general applications in immunology as well as in theoretical biological physics.

## March: Biophysics and biomechanics by Anders Hedenström

### How bats land head-over-heels

Flight in animals is energetically very costly, but because of its speed it can result in an overall relatively low cost of transport that allows birds, bats and insects to perform seasonal migrations. However, economic transport requires speeds where flight efficiency is high, allowing the wings to generate aerodynamic forces of high lift and low drag. A lot of research related to flight ecology has therefore concentrated on aerodynamic performance by animals, whereby popular models of animal flight often are applied to birds and bats as if they are interchangeable objects, despite their rather distinct flight morphologies [5].

**Fig 3:**
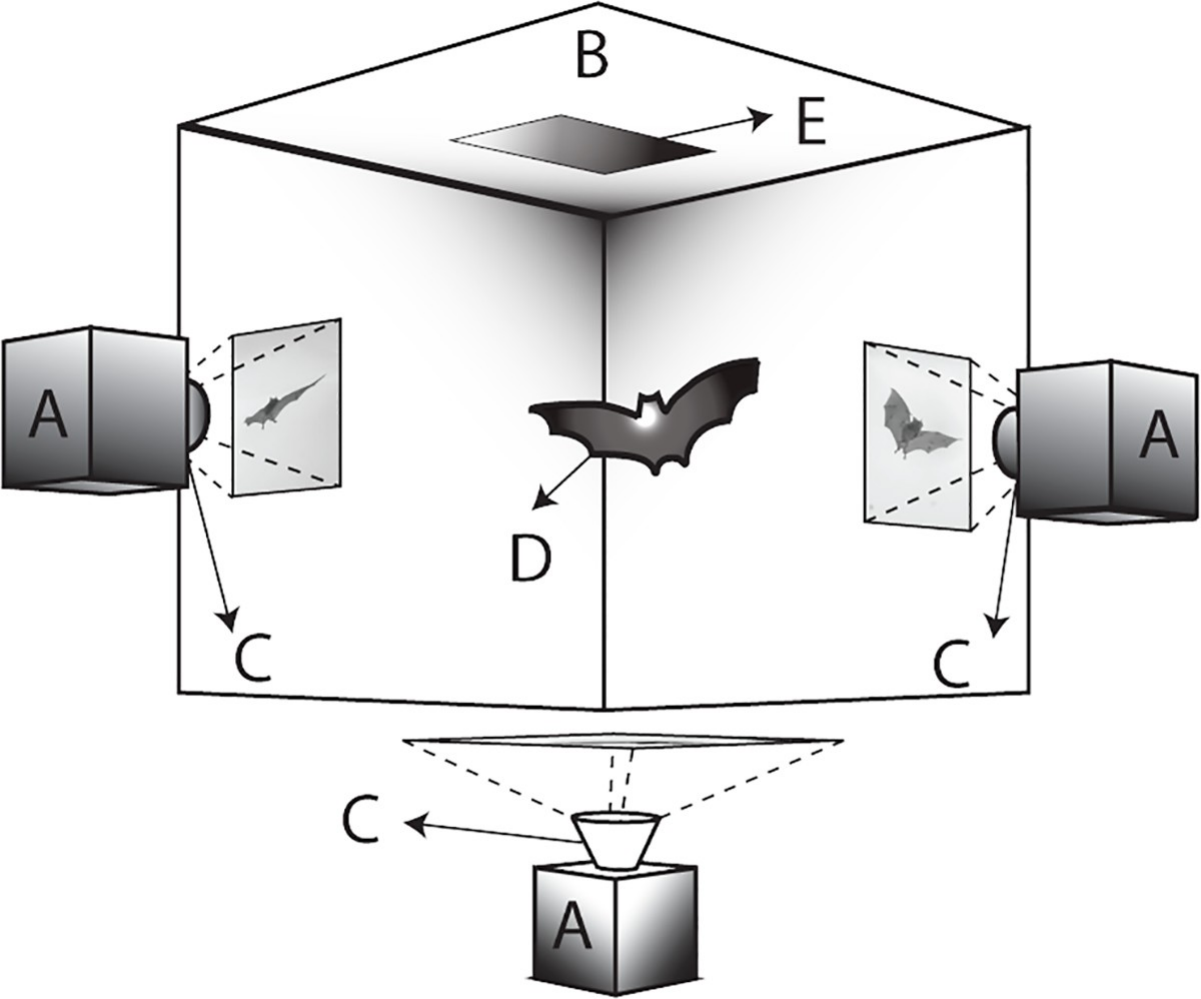
Apparatus used by Bergou et al. for their study. The three high-speed cameras (A), running at 1,000 frames per second, captured the motion of the bats as they landed on the ceiling pad (E). Image reproduced under CC-BY 4.0 license, credit: 10.1371/journal.pbio.1002297.

For the *PLOS Biology* XV Collection I have chosen to highlight an article by Bergou and co-workers [6] that focuses on inertial forces deployed by bats during their acrobatic approach to landing head-over-heels as bats usually do. In this study, Bergou at al. investigated how bats execute this acrobatic manoeuvre, exploiting asymmetric morphing of the wings to set up a torque that causes the body to rotate (Fig 3). To land on a surface the bat needs to get a grip with their tiny feet, which are interconnected to the wings via the inner wing membrane (the plagiopatagium) and therefore quite immobile. Because the bat has to slow down just before initiating the somersault, this prevents the use of aerodynamic forces–these are small anyway at such low speeds, and the proximity to the landing site prevents vigorous flapping.

**Fig 4:**
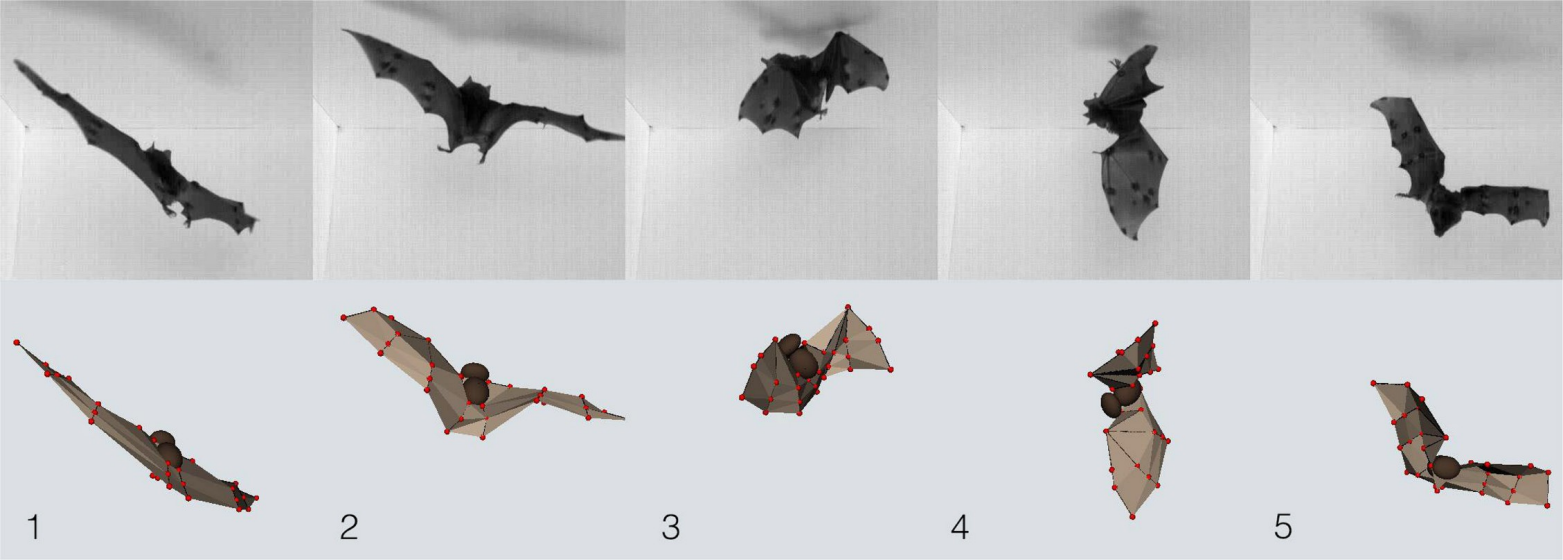
**Top row: Movie stills taken as a Seba’s short-tailed bat (*Carollia perspicillata*) tries–and fails–to land on the ceiling. Bottom row: 3D reconstruction of this activity.** Image reproduced under CC-BY 4.0 license, credit: 10.1371/journal.pbio.1002297.

Previous observations had showed how bats do it, but to dig further into how inertial forces replace aerodynamic forces, Bergoud et al. also analysed a simple model–a bat with rectangular wings and simplified kinematics, but still capable of generating manoeuvres similar to those seen in the real animal (Fig 4). The model was also extended to a “fully articulated model” with similar results.

The key to the inertia-related manoeuvres in bats is their relatively heavy wings, making it possible to set up a torque through asymmetric wing morphing. This may not be possible for other flying animals such as birds and insects, where wings are lighter than in bats. Another reason why inertia-related manoeuvres may not be useful to flies and hummingbirds is that they have also relatively stiff wings that prevent excessive morphing.

This article shows how complicated tasks such as everyday landing manoeuvres in bats consist of a rapid transition from aerodynamic-based cruising flight to an inertia-based somersault in free-fall under the ceiling. The study may also shed new light on our understanding of the evolution of flight, by investigating how incipient flight manoeuvres in a proto-flyer can be executed by using forelimbs with little or no aerodynamic lift.

## April: Microbiology by Michael Laub

### What happens when the antibiotic 1–2 punch backfires?

The rise of antibiotic resistance is a frightening reality. In response, many have insisted that we must find and develop new antibiotics. But developing even a single new antibiotic–one that is both safe and effective–is a daunting task that could take decades, if successful at all. Indeed, given the risks and the lack of financial incentives, many pharmaceutical companies have completely abandoned their antibiotic development efforts. This could be grim news with dire consequences for human health. But fortunately, there is evidence that we can deploy the current arsenal of antibiotics more effectively to circumvent resistance and the need for new antibiotics.

The article that I chose to highlight for the *PLOS Biology* XV Collection, “When the Most Potent Combination of Antibiotics Selects for the Greatest Bacterial Load: The Smile-Frown Transition”, tackles this issue [7]. In this article, Robert Beardmore and colleagues focus on understanding how combinations of antibiotics impact bacterial growth, with some rather counterintuitive results.

Combination therapy is a popular and powerful means of combatting many bacterial pathogens, viral infections, and even cancer. The simple, and perhaps simplistic, notion is that treating a bacterial infection with two antibiotics must be better than one. In particular, for two antibiotics that are initially synergistic, i.e. the combination suppresses growth more effectively than either does alone, it would seem logical that more is better. Continued treatment with those two antibiotics seems like the natural course of action to eradicate the bacteria. But what this article demonstrates is the exact opposite! What is initially a powerful combination can, in fact, lead to the highest load of bacteria in the long-run.

How do we make sense of such a counterintuitive result? Beardmore’s group showed that the key is to consider the competition that occurs between drug-susceptible and drug-resistant members of a population. Antibiotic-resistant mutants readily arise within almost any population of bacteria. If a population containing both susceptible and resistant mutants is treated with two antibiotics, the susceptible majority is, as expected, rapidly wiped out. But this also removes any competition for the resistant minority, enabling them to quickly grow and proliferate. In contrast, treating the same mixed population with a single antibiotic can be less effective initially as the susceptible bacteria aren’t eliminated as quickly, and their continued (albeit impeded) growth helps keep the resistant bacteria in check.

**Fig 5:**
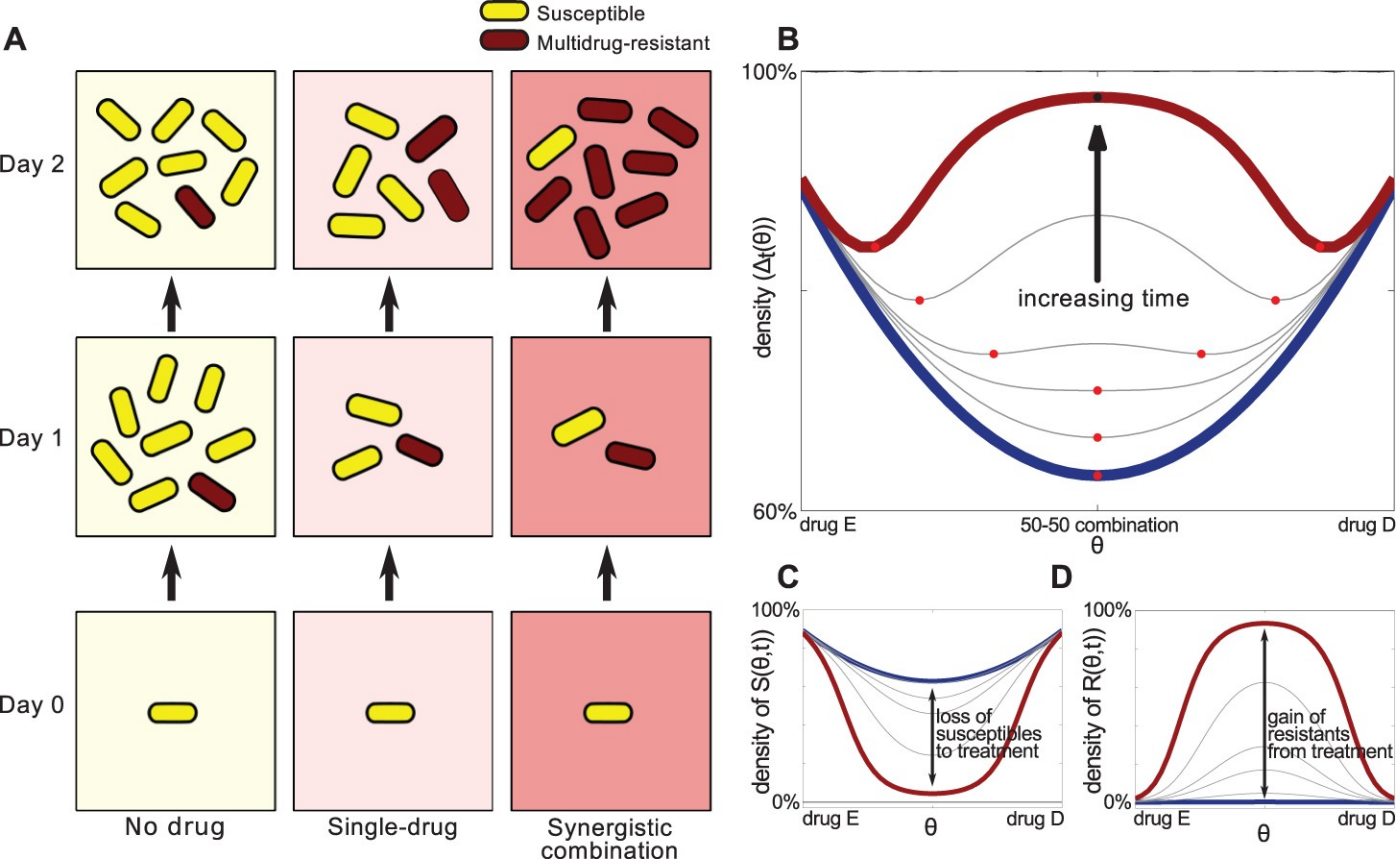
**A figure from Pena-Miller et al showing (A) how a synergistic combination of antibiotics can be initially optimal, but produce maximal growth in the long-term. (B) When the combinations range from monotherapy with one drug, to a 50:50 split, to a monotherapy with the other drug, the initial ‘smile’ gets flipped to a ‘frown’ over time.** Image reproduced under CC-BY 4.0 license, credit: 10.1371/journal.pbio.1001540.

This article demonstrates how such a competition-based model can, in principle, complicate the long-term dynamics of antibiotic-treated bacterial populations, leading to what they term a ‘smile-frown’ transition. To explain: if one plots the density of bacteria on the y-axis and various combinations of two antibiotics on the x-axis, ranging from a 100:0 split of antibiotic 1:antibiotic 2, to an even 50:50 split, to 0:100, the result is a ‘smile’, at least initially (Fig 5). But what the modeling suggests is that over time, this curve is inverted to a ‘frown’ as the 50:50 split goes from being the best combination to the worst.

The article demonstrates that this smile-frown transition occurs in real populations of *Escherichia coli*. And they show, as predicted from their modeling, that the transition depends on the emergence of antibiotic resistance, frequently through duplication of a drug efflux gene. Eliminating this gene largely eliminated the smile-frown transition.

This work by the Beardmore group is a beautiful demonstration of how easily our initial intuition can lead us astray and how complex dynamics can emerge in seemingly simple systems. Whether the exact principles learned from this article will apply in the context of clinically-relevant infections remains to be seen. But at a minimum, this article highlights the profound importance of careful and quantitative analyses in thinking about how we deploy antibiotic combinations. Such efforts might just be a better and more practical strategy in the coming era of antibiotic resistance than waiting for a new miracle drug.

## May: Ecology by Georgina Mace

### Effective conservation requires science-based decisions

The efforts made by conservationists to preserve vulnerable species and sustain critical ecosystem services face increasing challenges. Funding is limited, pressures on natural environments are escalating, and competing demands for the use of land and sea are intensifying. So how do conservation practitioners decide what to do with the limited resources available to them, and how might their actions be more efficiently designed?

The two papers that I have selected to highlight for the *PLOS Biology* XV Collection come from the Possingham group at the University of Queensland and introduce a formal basis for making conservation decisions [8,9]. The case studies in both these articles suggest that the most obvious actions may not be the best, and the authors present analytical approaches to improve decision-making. These approaches require a certain amount of scientific understanding of the system but applying these techniques to guide conservation actions will provide much better outcomes compared to relying on traditional wisdom.

The traditional approach for conservation is, first and foremost, to protect existing habitat. The alternative—habitat restoration—can be very costly, and recent experience reveals that the full suite of species and ecosystem services may be recovered only slowly over time. But even well-managed intact areas inevitably degrade over time due to growing anthropogenic pressures and environmental change. On the other hand, well-implemented restoration projects can deliver optimal conditions for certain species or ecosystem services. Rather surprisingly, these articles show that as restoration techniques improve, there are often circumstances where restoration should be prioritised over protection.

The studies use decision theory, employing resource allocation optimisation models given a fixed budget and a specific desired outcome. The articles are significant for putting conservation practice onto a more formal scientific and evidence-based footing. They are able to do this by taking a few specific steps that are not common practice in many conservation efforts, but perhaps should be.

**Fig 6:**
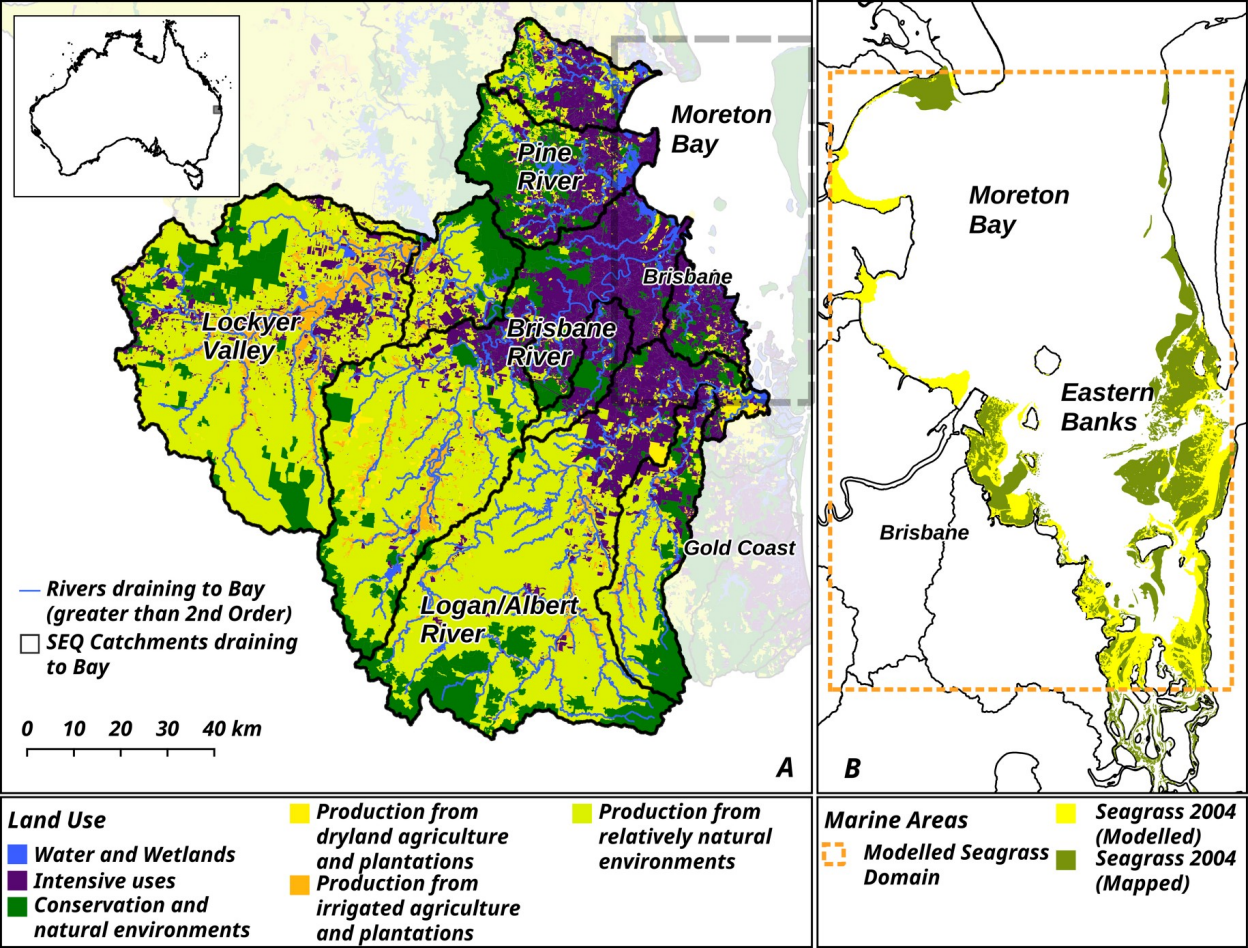
Map of the Moreton Bay area of Queensland, Australia, site of the seagrass case study in Saunders et al. Image reproduced under CC-BY 4.0 license, credit: 10.1371/journal.pbio.2001886.

The first critical step is to have a clearly defined outcome that is required for the area or habitat under consideration. Possingham et al (2015) [8] investigate two case studies. In the first, the objective is to maximise the storm protection services of intact mangrove ecosystems in the Coral Triangle, and in the second the objective is to minimise bird species extinctions in the Atlantic forest of Paraguay. The second article (Saunders et al. 2017) [9] investigates the more complex case of coastal ecosystems in Australia where the objective is to restore functional seagrass beds that are strongly affected by land-based sediment flows (Fig 6). Thus there are four choices for conservation actions for this system: restoration or recovery of land or ocean habitats. In each case study there is a fixed budget allocation over the next 30 or 40 years. The final input in each case is a dynamic and temporally explicit landscape or landscape-seascape model that integrates the costs and benefits of restoration or protection to find the optimal decision in each case. These three elements–an explicit objective, a fixed budget, and an effective model of the system are not often available, but when they are the results can be very influential.

In the mangrove case, restoration is favoured over protection because the storm protection service requires intact mangrove forest and restoration achieves a reduction in degradation more quickly, even though it is more costly. In the rapidly degrading Paraguayan forests, the optimal strategy is protection for the first 20 years, effectively reducing the rate area of degraded forest, but thereafter a switch to restoration achieves the greatest reduction in the number of bird extinctions. In the coastal study the surprising result is that the optimal strategy for restoring intact seagrass beds is restoration in the marine environment, and not addressing pollution sources on the land. This turns out to be more effective over the long term despite its higher costs. These conclusions are all sensitive to a number of input assumptions which are explored in the articles.

Crucially, the explicit optimisation models may not be possible in many real-world situations–they depend on substantial inputs from ecology and economics as well as practical experience. But both articles also use sensitivity analysis to explore different ecological contexts and provide simple rules of thumb to aid decision-making in practice. In real world cases there are often multiple objectives which complicate the analysis, but need not rule out adopting the approach.

Conservation practice is often far from evidence-based, but certainly should be. These articles provide a clear direction for the kinds of science and decision-making tools that could make a big difference. While the ecological and economic modelling is challenging, the identification of clearly stated quantitative objectives over reasonable time intervals need not be, and can be used along with the rules of thumb to inform better decision-making even under substantial uncertainties.

## June: Neurobiology by Piali Sengupta

### Re-interpreting pheromone signaling

Negative results that call into question a previously proposed model are generally difficult to publish unless they overturn a particularly high-profile finding. And yet, negative results are critically important for the advancement of knowledge; if a model isn’t supported by further experiments it must be reconsidered.

For the *PLOS Biology* XV Collection, I’ve chosen to highlight a 2013 article by Gomez-Diaz et al., “Ligands for pheromone-sensing neurons are not conformationally activated odorant binding proteins” [10]. This article experimentally addresses a previously published model for how a pheromone signals via its receptor in the fruit fly *Drosophila melanogaster* [11]. Negative data from Gomez-Diaz and colleagues indicated that this proposed model is likely incorrect.

Pheromones are small molecules that mediate intraspecific chemical communication and convey information about an individual’s social status, health, and sex, among others. In the individual that receives the signal, pheromones elicit long-term changes in physiology and development, as well as rapid changes in behavior. Decoding the complex language of pheromone signaling remains an ongoing challenge.

Pheromones play a particularly critical role in the lifecycles of insects. In *Drosophila*, the male-specific pheromone *cis*-vaccenyl acetate (cVA) regulates courtship and aggression behaviors. This pheromone is sensed via the Or67d olfactory receptor, which expressed in a subset of olfactory sensory neurons (OSNs) located in the T1 trichoid sensilla on the fly’s antennae.

Odorant-binding proteins (OBPs) are diverse proteins that facilitate the presentation of odorants to their cognate receptors, but aren’t thought to directly bind the receptors themselves. cVA signaling via Or67d requires an OBP called LUSH. Previous work had shown that in the absence of LUSH, basal spiking of Or67d-expressing OSNs decreased, leading to the hypothesis that LUSH may directly bind Or67d and thereby modulate OSN firing.

Laughlin et al [11] experimentally tested this hypothesis by generating a presumed constitutively active recombinant LUSH protein (LUSHD118A). They then infused this recombinant protein into the sensilla of lush mutant flies by including the recombinant protein in the recording pipet. This protein was found to dramatically increase spontaneous firing of the Or67d neurons; activity was not further increased upon cVA addition. These data were interpreted to support the notion that LUSH acts directly on Or67d.

Gomez-Diaz and co-workers used a different experimental approach to test this model. They generated transgenic flies expressing LUSHD118A, the same presumed constitutively active mutant, at endogenous levels under the control of lush regulatory sequences. The transgenic LUSHD118A did not result in the enhanced OSN activity that Laughlin et al. had observed in the infusion experiment. Moreover, these neurons retained the ability to be activated by cVA, and lush mutant flies expressing additional LUSH mutant proteins at endogenous levels didn’t recapitulate the increased or decreased cVA sensitivity shown by Laughlin et al.

What could account for this discrepancy? Recombinant LUSH protein purified from bacteria may have different properties than endogenously produced protein. Acute presentation of LUSH proteins at non-physiological concentrations may also have elicited non-physiological effects. Indeed, an independent transgenic strain expressing LUSHD118A [12] also failed to recapitulate the infusion results.

The negative results reported by Gomez-Diaz and co-workers indicated that LUSH is unlikely to directly activate Or67d in the manner proposed in previous publications. This article doesn’t include new experiments addressing LUSH function, and indeed the role of LUSH in pheromone signaling remains mysterious to this day. But by presenting these negative data, the work described by Gomez-Diaz et al is important as it rules out one potential hypothesis and allows the field to continue exploring exactly how pheromones collaborate with OBPs to elicit specific behaviors.

## July: Structural biology by Ann Stock

### Anatomy of a protein kinase spine and how to break it

The post-translational addition of phosphate groups to serine, threonine and tyrosine residues is a fundamental strategy for regulating protein activities in eukaryotes. Eukaryotic protein kinases—the enzymes that catalyze these modifications—are critical to cellular function, and aberrant kinase activities are associated with many diseases including cancer, inflammation, infection, diabetes, hypertension, and neurodegeneration.

Eukaryotic protein kinases are therefore important targets for therapeutic intervention, now constituting a quarter of all drug discovery and development efforts, and ranking second only to G-protein-coupled receptors (GPCRs) as pharmaceutical targets. More than three dozen kinase inhibitors have received FDA approval since 2001, when Imatinib (Gleevec), an inhibitor of bcr-abl kinase, was approved for treatment of chronic myelogenous leukemia (CML). Rational drug design has played an essential role in kinase inhibitor development, with molecules being designed to target specific kinase conformations. Understanding the structural basis for regulation of protein kinase activity is therefore key to these efforts.

For the *PLOS Biology* XV Collection, I’ve chosen to highlight an article from the laboratory of Susan Taylor [13] that defines a set of intramolecular interactions that distinguish inactive and active conformational states of eukaryotic protein kinases. Such classification is complicated, because unlike many enzymes, eukaryotic kinases do not have single discrete active and inactive conformations, but instead are dynamic, with multiple conformations populating the two functional states.

**Fig 7:**
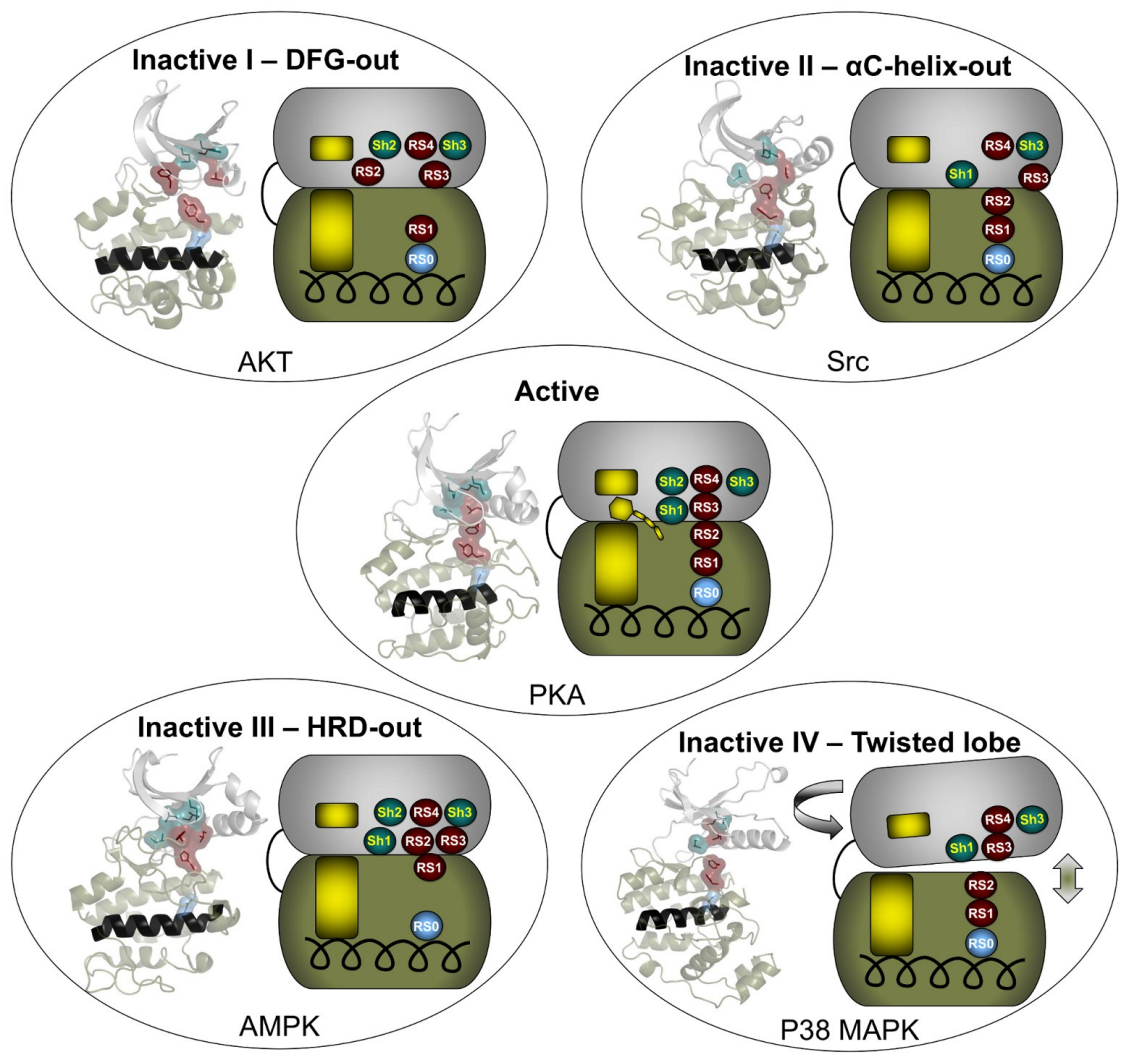
A figure from Meharena et al. showing the R-spine configuration in the active state (center) and four different inactive conformations. **Cartoon depictions of representative kinase structures are shown alongside schematic representations of the N- and C-lobes, with the C-spine depicted in gold, R-spine residues depicted in burgundy, and Shell residues in teal.** Image reproduced under CC-BY 4.0 license, credit: 10.1371/journal.pbio.1001680.

The catalytic core of eukaryotic protein kinases consists of conserved N- and C-lobes with the active site located at the interface of these two lobes. Previous studies had identified three hydrophobic features in the catalytic core: the αF-helix in the C-lobe and two clusters of non-contiguous residues in the primary sequence that coalesce in the three-dimensional structure to form two hydrophobic “spines” that span the N- and C-lobes. The Catalytic (C) spine includes the adenine moiety of bound ATP, which bridges hydrophobic residues in the N- and C-lobes. The Regulatory (R) spine, which typically consists of two aromatic residues in the C-lobe (RS1 and RS2) and two aliphatic residues in the N-lobe (RS3 and RS4), runs parallel to the C-spine, is aligned in a contiguous hydrophobic patch in the active state, and is disassembled in the inactive state (Fig 7).

Meharena and colleagues examined R-spine residues in the sequences of ~13,000 kinases and tested hypotheses about the nature of these residues by mutational analyses of the representative kinase cAMP-dependent protein kinase (PKA). They observed that residues RS1 and RS2 in the C-lobe were extremely sensitive to mutation, in contrast to the relative robustness of residues RS3 and RS4 in the N-lobe. This led to the identification of a set of three highly conserved residues that surround RS3 and RS4. These residues that support the R-spine in the N-lobe were dubbed Shell residues Sh1, Sh2 and Sh3. Additional mutational analyses provided experimental validation of the hypothesis that integrity of the R-spine is essential for catalysis. Furthermore, the data provided evidence that phosphorylation of the activation loop promotes activation by stabilizing the R-spine.

Knowing that an assembled R-spine is required for an active state, the group examined available structures of eukaryotic protein kinases and identified 172 structures in which R-spines were disassembled. They were able to classify four specific ways in which the R-spine was broken. Two of these correlated with previously characterized inactive conformations associated with positioning of the DFG motif in the activation loop. One conformation involves a DFG-out orientation, and the other, a DFG-in orientation caused by movement of the αC-helix. These two inactive conformations have already been successfully targeted for drug development.

The description of additional inactive conformations provides opportunities for new strategies for drug design and a broader foundation for interpreting and perhaps eventually modulating the molecular defects caused by protein kinase mutations associated with human disease.

## August: Infectious diseases by Andrew Read

### Perverse outcomes of novel therapies

Yale professor Steve Stearns once warned that the transition from Young Turk to Old Turkey happens quickly. He was right. Being an Old Turkey has challenges, not least that you more readily spot ignorance but you have less bandwidth to think it through. So, it is a total joy to come across an analysis you wish you’d had time to do yourself. The article I have chosen for the *PLOS Biology* XV Collection is one of those. In a 2014 Essay [14], three Young Turks—Pedro Vale, Andy Fenton and Sam Brown—considered a strategy said by others to be a solution to one of the great health challenges of the 21st century. Before their article, all I could see was a jumble of plusses and minuses. They sorted that out.

**Fig 8:**
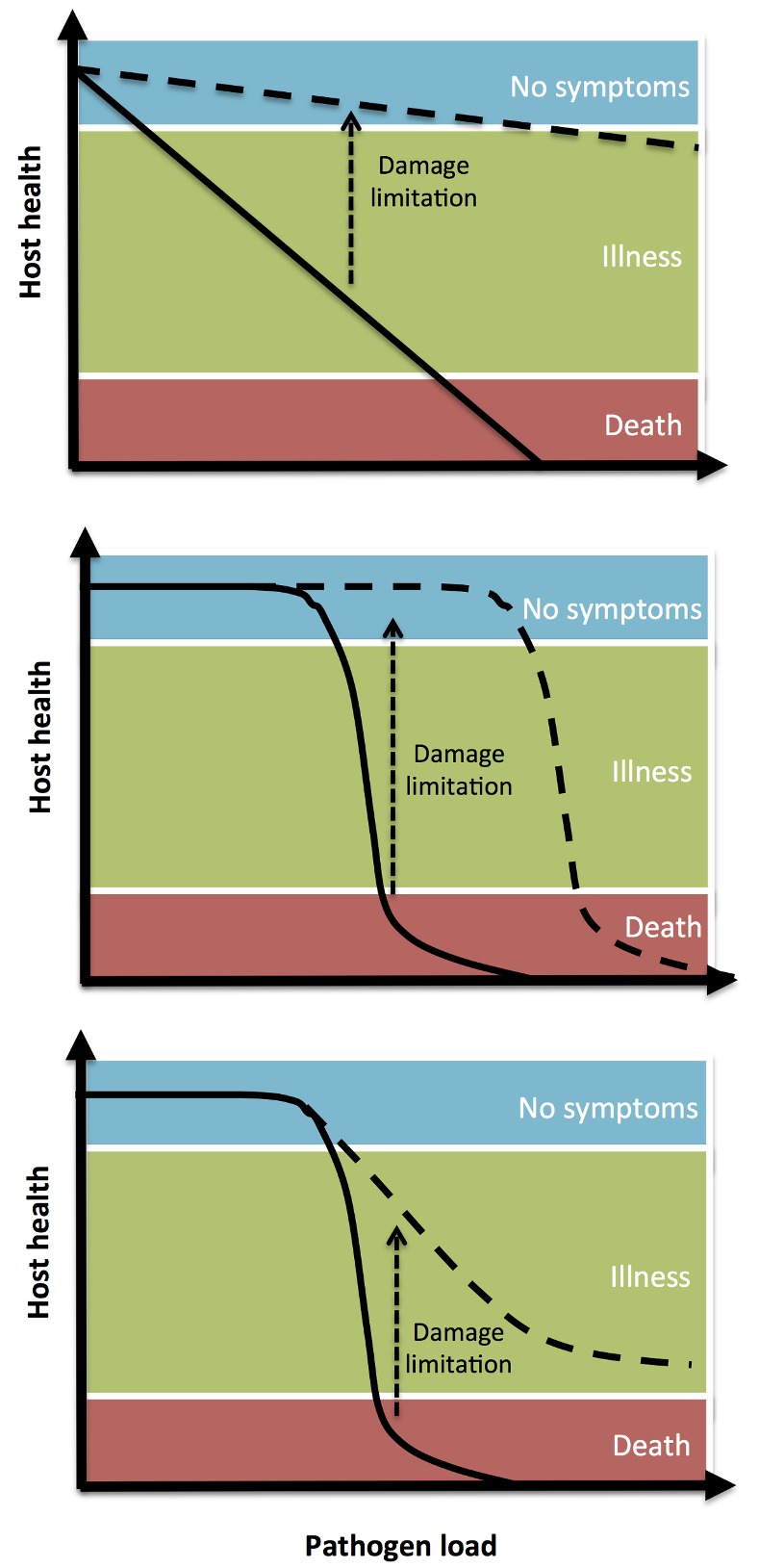
The effect of damage limitation mechanisms on the loss of host health during infection. **One can imagine several relationships between increasing pathogen load and host health, which may be infection- or pathogen-specific.** Image reproduced under CC-BY 4.0 license, credit: 10.1371/journal.pbio.1001769.

Prompted by the antimicrobial resistance crisis–said by some to be a bigger threat to humanity than terrorism–the search is on for drugs that can treat patients infected with resistant bugs–ideally without causing resistance themselves. One idea is to find drugs which make bugs less lethal (for example, by inactivating secreted bacterial toxins) or by making the patient more robust (for example, by enhancing tissue repair). The thinking is that such drugs would make the patient feel better and–because the bug is allowed to survive–resistance evolution won’t ensue (Fig 8).

The claim that these ‘damage-limitation’ drugs would be evolution-proof set off alarm bells in my head when I first heard it soon after the turn of the century. The only thing that stops evolution is death. Otherwise, as actor Jeff Goldblum put it in Jurassic Park, “Life, uh, finds a way.” Vale et al. provide what I consider the first sensible analysis of the epidemiological and evolution consequences of damage-limiting drugs. In a few pithy paragraphs (and seven lines of algebra), they show there are many possible consequences, not all of which are good. Most immediate are the transmission consequences. Not only will bugs be left alive to transmit, but infectious people will be harder to spot and, being less sick, more likely to be in contact with susceptible people. And the evolutionary outcomes also need not be good. For instance, if toxin production gives pathogens fitness advantages, as it almost always does, inactivating toxins with drugs might select for bugs that produce more toxin than the drugs can deal with–or bugs that can produce other toxins. None of those potentially harmful outcomes have to eventuate, as Vale et al. make clear, but they very well might.

The risk of harm is not an argument against developing damage-limiting drugs. But it is a strong argument for not considering them as magic bullets. Just like conventional drugs, their impact on pathogen transmission and evolution needs to be studied at all stages of the discovery pipeline–and post-roll out. Vale et al. provide the roadmap for things to look for. It’s an article I wish I’d written.

## September: Evolutionary biology by Harmit Malik

### Living l’AVIDA loca—origins of multicellularity

Even before John Maynard Smith formalized the term, evolutionary biologists have been fascinated by major evolutionary transitions, including the transition from single-celled to multicellular organisms. Multicellularity occurs over and over in the evolutionary record, giving rise to the bounty of life-forms visible to the naked eye. Simple, undifferentiated multicellularity can be easily explained as an example of practical cooperation, wherein different single-celled entities pool resources in a multicellular commune while preserving their individuality and the right to procreate.

However, differentiated multicellularity involves a Faustian bargain, akin to eusocial insect societies with few queens and many workers. What gives rise to such reproductive division of labor between differentiated non-reproductive cells that make up the soma and the few reproductive germ cells that are capable of giving rise to new offspring? The ‘dirty work’ hypothesis proposed that the pressures of doing ‘work’ (cell division, metabolic activity) are inherently mutagenic, and therefore likely to compromise genomic integrity. Thus, sequestration of cells dedicated to the task of reproduction absolves them of ‘dirty work’ which keeps their genomes as pristine as possible, and thus leads to the germ-soma dichotomy.

Despite being elegant and powerful in its simplicity, the ‘dirty work’ hypothesis is nevertheless tricky to test since it would require capturing the transition to differentiated multicellularity in the act. In recent years, experimental evolution models have made significant advances in our understanding of transitions to undifferentiated multicellularity in many unicellular organisms (yeast, *Volvox*, *Chlamydomonas*, choanoflagellates) but reproductive differentiation remains elusive. Enter AVIDA, a powerful artificial life software platform designed by Charles Ofria, Chris Adami, and C. Titus Brown, to study the evolutionary biology of self-replicating and evolving digital cells, self-replicating computer programs that evolve in an open-ended fashion.

**Fig 9:**
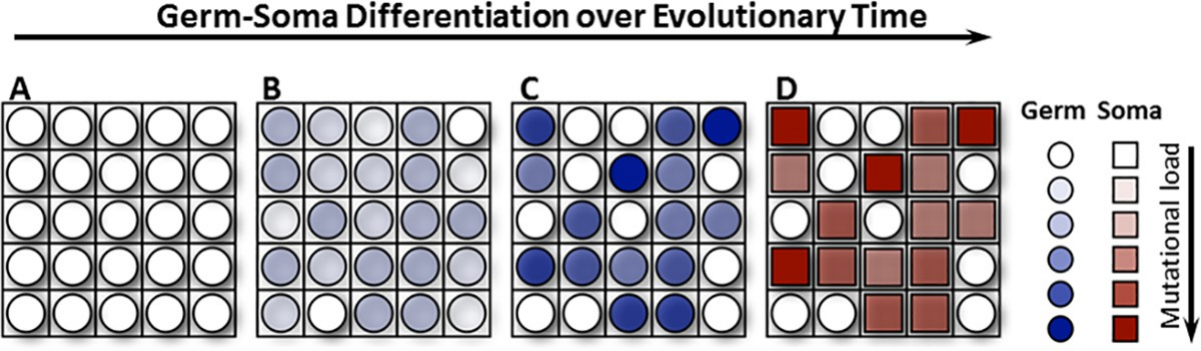
**Over evolutionary time, multicells change from consisting entirely of germ cells (A) to consisting of a blend of germ and soma cells (D), where germ cells serve as propagules (founders for a new multicell) and soma cells perform the mutagenic work. (A) Germ cells do not perform mutagenic work. (B) Germs cell do perform mutagenic work. (C) A subset of germ cells performs mutagenic work. (D) Soma cells, but not germ cells, perform mutagenic work.** Image reproduced under CC-BY 4.0 license, credit: 10.1371/journal.pbio.1001859.

In a fascinating article published in 2014 in *PLOS Biology*, Heather Goldsby and colleagues created a world of 400 multicells in the AVIDA platform [15] (Fig 9). Each multicell is akin to a multicellular organism, and comprises individual digital cells, performing a combination of logic functions, which ultimately determines how many resources flow into the multicell, and thereby its ability to replicate. Into this system of multicellular ‘organisms,’ the authors introduced a mutation rate into individual cells, which allowed them to acquire new functions and therefore more resources but also increased the probability of ‘genomic’ damage. Intriguingly, as the authors vary the mutation rate, they find ‘somatic’ cells incapable of self-replication at intermediate mutation rates. Closer examination confirms that these somatic cells are indeed performing more metabolic functions (‘dirty work’) and therefore taking a bigger hit to their genome integrity, whereas ‘germ’ cells maintain their own, and therefore the multicell’s, genetic information in pristine condition.

The authors exploit the power of AVIDA by following individual lineages of multicells (and their constituent cells) to find that multicells almost always first favor the development of ‘pseudo-somatic’ cells that perform much higher metabolic functions and therefore increase organismal (multicell) fitness. These ‘pseudo-somatic’ cells subsequently lose their ability to self-replicate, thereby avoiding a catastrophe of genomic mutation in the organism. Thus, a division of labor between ‘dirty work’ and reproduction is the most successful long-term strategy for multicellular organisms. Indeed, acquisition of somatic cells also allowed multicells to explore previously forbidden phenotypic niches under conditions in which mutational load increases with every new function. One can easily imagine that an increase in body size and complexity may represent such a mutational ramp-up. Another elegant article in *PLOS Biology* [16] nicely demonstrates that preservation of mitochondrial genome integrity and quality is one example of how germ cells in a multicellular organism may avoid the cost of ‘dirty work’ paid by the soma.

Despite involving digital life-forms, experiments such as those performed by Goldsby et al. are an excellent example of the extraordinary insight they provide into evolutionary transitions that might otherwise be impossible to study with living, biological organisms. Cooperative systems such as multicellular organisms always operate under peril of cheaters, mutation-laden somatic cells that do not cede the reproductive role to pristine germ cells. However, the authors show that multicellular organisms comprising these high-mutation cells are less fit. Thus, their experiments not only provide valuable insight into ancient evolutionary transitions to multicellularity but may also guide studies of reversion to unicellularity, whereby cancerous cells arise by flouting rules governing replication or quiescence in multicellular organisms.

## October: Plant biology by Mark Estelle

### Auxin transport—more a river delta than a stream

The sessile lifestyle of plants is enabled by remarkable developmental plasticity. Plant form is affected by a wide range of environmental conditions from nutrient availability to herbivory, so two plants with the same genotype can appear quite different, depending on their environment. All plant organs and tissues are derived from dispersed stem cell populations called meristems. Individual meristems can act independently to a certain extent but are also controlled by systemic signals that coordinate and integrate their activities. The identity of these signals and how they function, has long been a subject of intense interest to plant biologists. The article that I chose to highlight for the *PLOS Biology* XV Collection is by Tom Bennett, Ottoline Leyser and colleagues and provides important new insight into the nature of systemic communication [17].

One of the best systems for understanding how plant development is coordinated is the behavior of meristems that are found in the axil of each leaf. These axillary meristems often persist in a dormant state, as buds, until such time as they are activated. For example, if the shoot tip is removed by herbivory or with a pair of scissors, bud dormancy is released, and a branch is produced. This phenomenon, called apical dominance, depends upon movement of the plant hormone auxin from the apex of the plant down through the stem. It is well known that auxin moves via a specific transport system called the polar auxin transport stream or PATS. PATS acts to rapidly move auxin through files of cells in the stem, specifically the xylem parenchyma and vascular cambium, from the plant apex down into the root system. Auxin transport requires one of a family of auxin cellular efflux carriers called the PIN-FORMED (PIN) proteins. Polar transport is the result of localization of the PIN1 transporter to the basal side of the cell.

**Fig 10:**
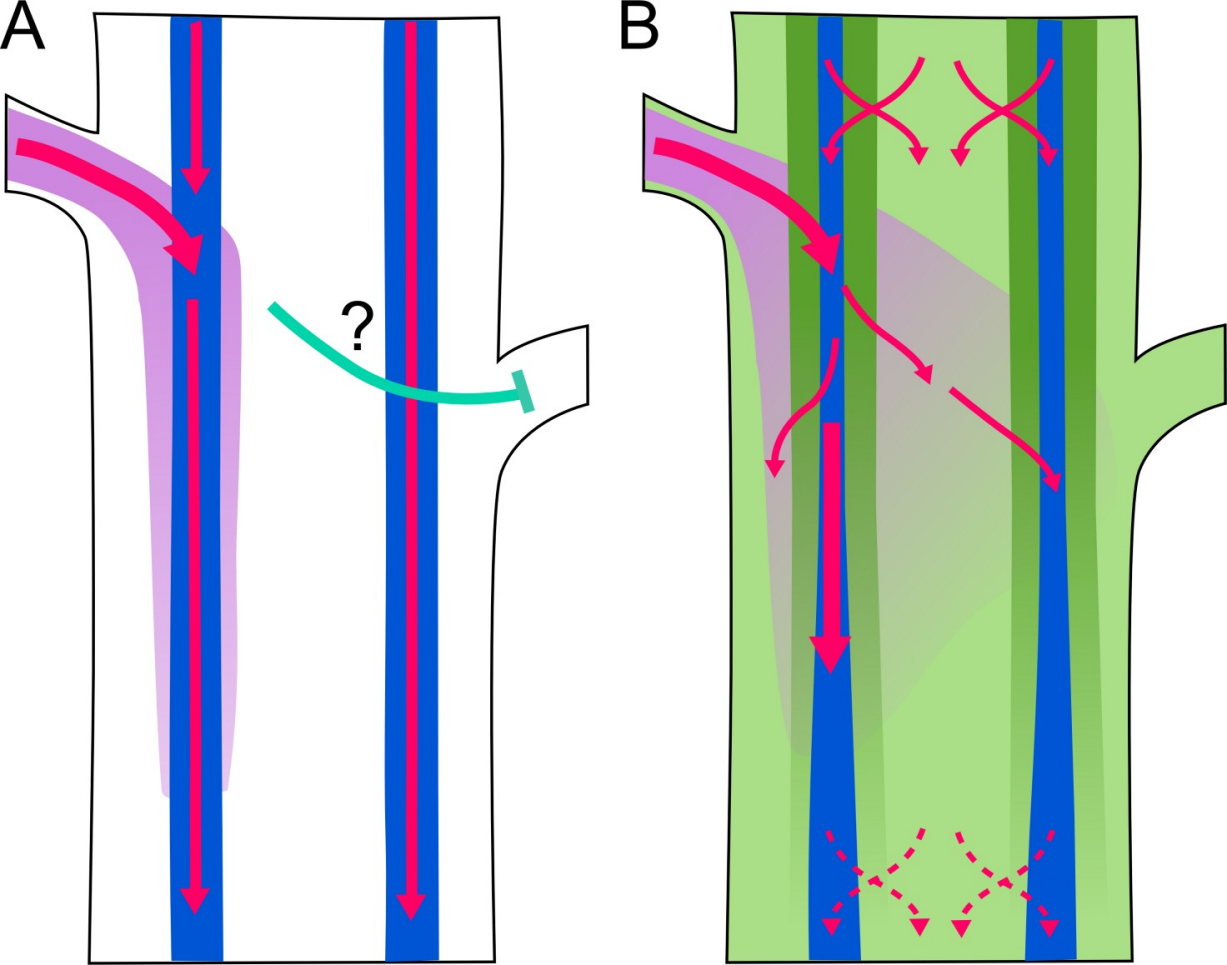
Two proposed models of auxin transport in an Arabidopsis stem. Image reproduced under CC-BY 4.0 license, credit: 10.1371/journal.pbio.1002446.

Over the last decade, the Leyser group has performed elegant studies to explain how PATS regulates shoot branching (Fig 10). For bud activation to occur, a PATS must be established that transports auxin from the bud into the existing PATS in the stem. This appears to happen through a canalization process in which passive auxin transport from a source to a sink, upregulates and polarizes PIN transporters leading to formation of narrow polarized transport stream. Whether or not this happens depends on the source-sink relationship between the bud and the stem PATS. If auxin levels in the PATS are high, it will be a weak sink and transport from the bud will not occur. In contrast, if auxin levels are low, as is the case after decapitation, the stem PATS will be a strong sink, leading to establishment of a new PATS from the activating bud into the stem. It’s important to note that the PATS is sensitive to many other factors besides decapitation (a dramatic environmental stimulus) including light, nutrient availability and the genetic program. In this way the PATS functions as a central integrator of shoot branching control.

Although both experimental and mathematical modeling approaches support this model, it does not explain all aspects of branching regulation. For example, buds on opposite sides of the stem will transport auxin into different vascular bundles, yet still inhibit each other’s activation. To try to explain this sort of behavior, Bennett et al. explored the possibility that other auxin transport streams may exist in the stem. In particular, they investigated the role of other members of the PIN family in branching regulation. Their results indicate that auxin transport is much more complex than previously proposed. In addition to PIN1-based highly polar transport, PIN3, PIN4, and PIN7 contribute to widespread and less polar auxin transport, which they term “connective auxin transport” (CAT). Modeling studies demonstrate that CAT provides important local information that helps to coordinate the behavior of axillary meristems that are close together.

This work is extremely satisfying because it takes us beyond the simplistic view of polar auxin transport that has dominated the field for decades to a more complex understanding of how meristems communicate through local and long-distance movement of auxin.

## November: Developmental biology by Sally Lowell

### A spotlight on spottiness

Let’s start with a remarkable fact. Cells can, under the right conditions, organise themselves into patterns without any outside instruction. Indeed, it is the ability of cells to self-organise that makes multicellular life possible. Contemplating this, it soon becomes apparent that the only proper course of action is to become a developmental biologist and devote one’s life to trying to understand how such things can possibly happen.

This phenomenon captured the imagination of mathematician and code-breaker Alan Turing, who famously described one mechanism for the spontaneous emergence of periodic patterns. In a “Turing mechanism” the initially uniform secretion of a diffusible ‘activator’ molecule triggers the production of a faster-diffusing ‘inhibitor.’ Over time, instabilities in the system become amplified until these molecules resolve into patterns. Examples of such patterns might include the stripes of a tiger or the spots of a leopard. Even non-leopards such as you and I have a spotty distribution of hair follicles in our skin, following a pattern that could be explained by a Turing mechanism.

In the article that I’ve chosen to highlight for the *PLOS Biology* XV Collection, Glover et al [18] set out initially to identify the components of the putative Turing mechanism that patterns hair follicles. The authors used a beautiful live explant system that allowed them to follow patterning in real time. They successfully homed in on a signalling network that seemed to be sufficient to explain the distribution of hair follicles. They then observed that the first ‘pre-pattern’ emerges within the epidermal layer of the skin, and this then dictates the position of mesenchymal condensates—groupings of mesenchymal cells necessary for the formation of a new hair follicle—in the underlying dermis.

**Fig 11:**
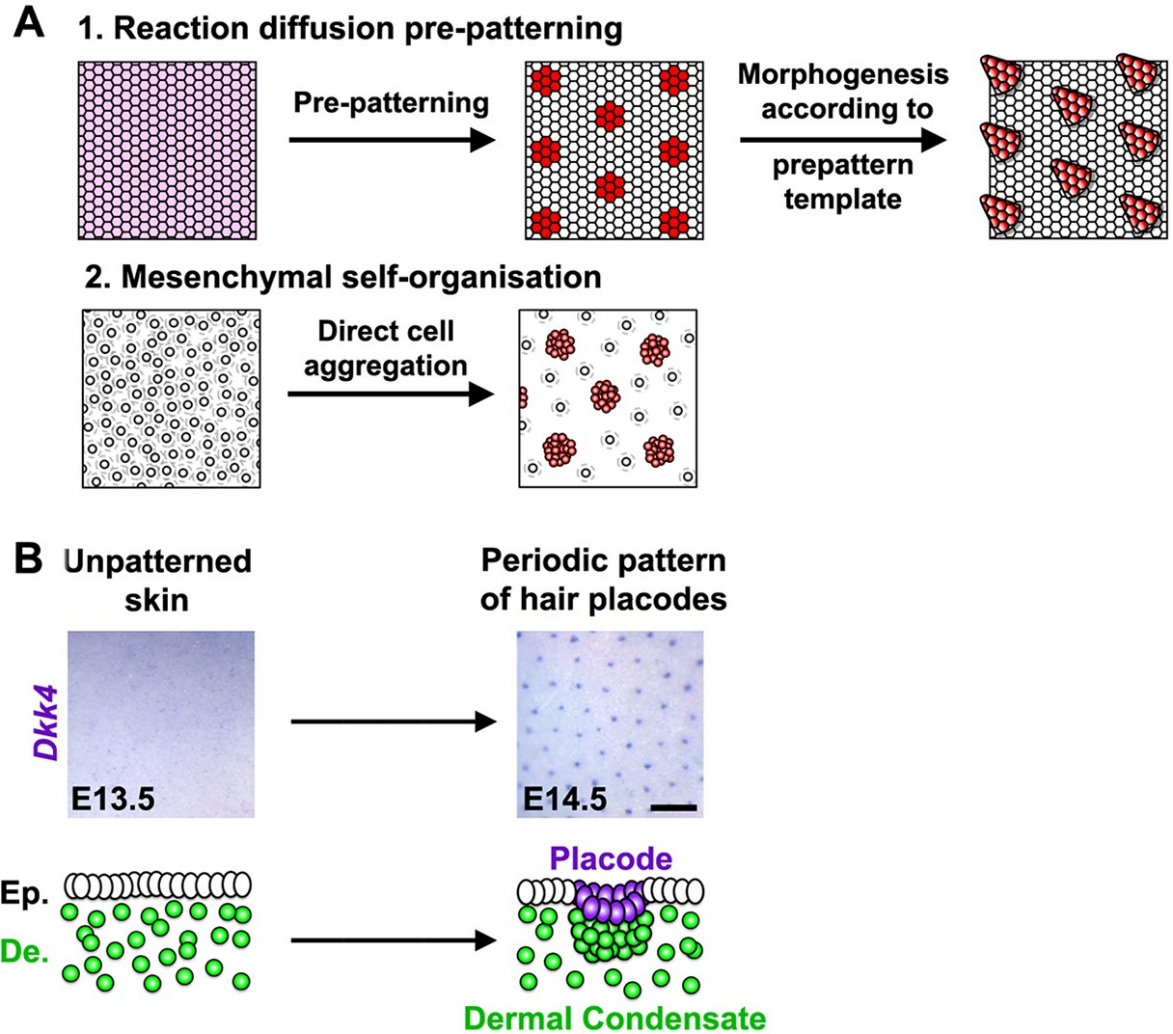
Two models explaining the emergence of repeating patterns and a schematic of the process of hair follicle formation. Image reproduced under CC-BY 4.0 license, credit: 10.1371/journal.pbio.2002117.

So far, so good: the pattern is explained. Now comes the surprise. Because the authors now knew which particular signals drive Turing patterning, they were able to disrupt the distribution of these signals and show that this wipes out the epidermal prepattern. Unexpectedly, a suitably spotty distribution of condensates still somehow emerged in the dermis. At first this looked very similar to the usual Turing pattern, but Glover et al noticed a few tell-tale differences. Instead of lining up neatly along cut edges of explants, spots now seem to avoid sitting too close to these edges. The dynamics of patterning was also altered. These careful observations, combined with modelling, suggested that this epidermis-independent spottiness was driven not by Turing patterning (which you’ll remember is driven by diffusion of activator and inhibitor molecules) but by a conceptually similar but mechanistically distinct mode where it is cells rather than molecules that move. The authors found that patterning is driven through local aggregation of cells. This clustering becomes reinforced locally as cells draw closer to each other and is inhibited more distantly because cells become sparser as they move away from the future interfollicular regions and towards the aggregates (Fig 11). Glover et al went on to identify the molecules that mediate this previously-cryptic mesenchymal self-organisation process and to reveal how it is linked with epidermal Turing patterning in a hierarchical process.

These findings have implications beyond explaining our hairiness. They make us look with a fresh eye at other patterning processes and wonder whether there may be additional cryptic mechanisms lurking undiscovered behind our textbook models. For example, it has long been known that patterning at gastrulation is dictated in mammals by signalling centres in the extraembryonic tissues, so it came as a shock when it was recently discovered that similar patterns somehow still emerge within aggregates of pluripotent cells even in the absence of extraembryonic tissues [19–22]. Are there two distinct but interlinked mechanisms operating at gastrulation, just as there are in the skin?

The study raises a number of other questions. Is the cryptic “secondary” patterning mechanism merely a remnant of evolutionary history, or could it be important for ensuring that patterning is robust? Could the principle of using multiple interacting patterning mechanisms be useful in guiding efforts to engineer patterns into groups of cells [23]? Perhaps the broadest lesson here is that we should not rush to decide between apparently competing hypotheses on the assumption that one of them must be wrong. Sometimes biology really does let us have it both ways.

## December: Meta-research by Jonathan Kimmelman

### Ethical oversights in ethical oversight of animal research

Sometimes the life sciences work fantastically, as when insights into fundamental processes are transformed into life-saving treatments. Other times the scientific process flops: false claims take on a life of their own [24,25], or ineffective treatments are advanced into drug development [26] and/or care [27]. A key to improving the balance of successes versus failures is the systematic investigation of how science works—a line of inquiry known as meta-research. *PLOS Biology* is the only general life science journal to offer strong support for this project by devoting a specific section to meta-research reports.

Among the many excellent meta-research articles published by *PLOS Biology*, I’ve chosen to highlight Vogt et al [28] for the XV Collection. It stands out for offering a glimpse of research evaluation processes that are all but inaccessible to systematic analysis because they typically occur behind the closed doors of institutional review panels.

First some background: since roughly the 1970s, various government authorities have required that research proposing to use nonhuman animals undergo an independent review and approval process before it is conducted. Such review processes stem from the ethical sensitivities surrounding experiments that use nonhuman animals as their research reagents. Unlike rocks, chemicals in the Sigma catalogue, or subatomic particles, animals have a capacity for suffering and their interests must be protected. Animal care committees are charged with making an independent judgement about whether nonhuman animal studies are morally justified. Yet the documentation submitted to these committees is deemed confidential and little is known about precisely what factors drive those judgements.

In Vogt et al, the authors accessed almost 1300 applications to conduct animal experiments in Switzerland, a jurisdiction where reviewers on animal care committees are instructed to weigh an experiment’s harms against its potential benefits. Outside of Switzerland, access to such protocols is almost impossible; indeed, researchers often aren’t even required to provide a detailed research protocol.

The authors then examined the extent to which these application protocols described the implementation of practices aimed at addressing threats to the internal validity of experiments. These practices are important safeguards against faulty experimental design and researcher bias. Vogt et al found that few applications stated an intention to use procedures like randomization or concealed allocation. Such practices were only slightly more common for studies involving “higher” nonhuman animal species like cats, dogs, and primates. But even here, they were uncommon. For example, fewer than 20% of such studies proposed a blinded study design. The authors conclude that animal care committees in Switzerland approve experiments based not on an appraisal of the scientific methods, but rather on confidence, based perhaps on the scientific bona fides of researchers and/or sponsors.

Regretfully, the lack of rigorous evaluation of proposed research seems to extend to human research ethics committees as well. In 2018, *PLOS Biology* published an article by Wieschowski et al examining the preclinical justification for 106 early phase clinical trial protocols submitted for institutional review boards (IRBs) at German institutions [29] (disclosure: I am a middle author on this publication). The report found that 17% of protocols did not cite any preclinical efficacy studies. Those protocols that did cite preclinical efficacy studies offered scant information on the extent to which such studies had addressed various threats to clinical generalizability. One logical implication of this report is that—as with nonhuman animal studies in Vogt et al—ethics review committees approve early phase trials without a clear appraisal of their evidentiary grounding. Instead they rely on confidence in the researchers, sponsors, or perhaps other regulatory processes.

Scores of studies have previously documented deficiencies in the methods described in preclinical study publications. What makes Vogt et al stand out from these other studies, however, is that such deficiencies are documented farther upstream—at the point where studies are designed and reviewed.

Vogt et al (and Wieschowski et al, too) has other, more profound implications. Nonhuman animal and human experiments may look as if they are entirely conceived of and designed by scientists and research sponsors. Yet IRBs and animal care committees—far from mere bureaucratic after-thoughts—play a critical role in shaping what questions are asked in research and how such questions are resolved. Among other things, such committees grant scientists the moral license for pursuing research that might otherwise be deemed inhumane or unethical. In so doing, they signal to scientists and others what sorts of research practices are proper and which ones are not, and scientists who want to get their protocols approved quickly learn to internalize these norms.

Yet such committees process large volumes of highly technical research protocols and must rely on heuristics for assessing the relationship between a study’s burden and its value. Whether that is “confidence”” (as alleged by Vogt et al) or precedent, it’s hard to avoid concluding that many aspects of ethical review in life science research contradict the spirit of independent, systematic and unbiased risk/benefit analysis enshrined in various policy documents. If the life sciences suffer from an excess of unreproducible findings, the ethical oversight (in both senses of the term) is partly to blame.
